# Supplementary material for: Single cell and spatial transcriptomics highlight the interaction of club-like cells with immunosuppressive myeloid cells in prostate cancer
Source: Nat Commun. 2024 Nov 16;15:9949. doi: 10.1038/s41467-024-54364-1 (PMC11569175; doi:10.1038/s41467-024-54364-1)
Supplement: Supplementary file 2 — Description of Additional Supplementary Information [file 41467_2024_54364_MOESM2_ESM.pdf]

Supplementary Data Legend

Supplementary Data S1: Discovery (sheet 1) and validation (sheet 2) cohort patient characteristics and spatial transcriptomics sample metrics.

Supplementary Data S2: Single-cell RNA-sequencing data-derived gene signatures (sheet 1) and source dataset information (sheet 2).

Supplementary Data S3: Inferred cell state counts across spatial locations (sheet 1) and split by sample category (sheets 2-5).

Supplementary Data S4: Overexpressed genes in single-cell mapping-derived regions (SCM regions) by sample. Each sheet contains results for all genes overexpressed (adjusted p-value < 0.05 and log-fold change ≥ 1) in the region (sheet name) of a particular sample when compared against all other locations in that sample.

Supplementary Data S5: Single-cell mapping-derived region (SCM region) marker gene overrepresentation analysis results. Each row is a gene entry and includes the number of samples in which the gene was overexpressed in the region (sheet). Each genes' overrepresentation as a marker for the given region was tested using a one-sided Fisher's exact test and the p-value adjusted for multiple testing is shown.

Supplementary Data S6: Differential gene expression analysis results for the Club regions of BPH, TRNA, NEADT, and CRPC sample categories. Each sheet is named according to the comparison in question (test_vs_control).

Supplementary Data S7: Gene sets (sheet 1) used in calculating the gene set activity scores, including a source reference (sheet 2).

Supplementary Data S8: Enrichment analysis results between region-specific gene markers and gene sets. Rows are gene sets and columns are regions (sheet 1). Entries are one-sided Fisher's exact test p-values adjusted for multiple testing. For each region, the overlap between region markers and significantly enriched gene sets is shown (sheets 2-9).

Supplementary Data S9: Ligand-receptor binding analysis results in the Club region interfaces. Each row is an unique ligand-receptor pair, containing the interacting region, fraction of interfaces in which the ligand-receptor interaction was active ($p_{adj} < 0.05$). The total number of interfaces is also displayed. Each ligand-receptor interaction was tested for enrichment in the interface and a one-sided Fisher's exact test p-value adjusted for multiple testing is displayed.

Supplementary Data S10: Metastatic tumor sample patient characteristics and spatial transcriptomics sample metrics (sheet 1), and differential gene expression testing results between the clusters within each sample (sheets 2-5).

Supplementary Data S11: Differential gene expression analysis results of pseudo-bulked (concatenated ST data counts) expression between neoadjuvant-treated (test) and treatment-naïve (control) samples.
