# Supplementary material for: Single cell and spatial transcriptomics highlight the interaction of club-like cells with immunosuppressive myeloid cells in prostate cancer
Source: Nat Commun. 2024 Nov 16;15:9949. doi: 10.1038/s41467-024-54364-1 (PMC11569175; doi:10.1038/s41467-024-54364-1)
Supplement: Supplementary file 14 — Reporting Summary [file 41467_2024_54364_MOESM14_ESM.pdf]

nature portfolio

Corresponding author(s): Matti Nykter, Alfonso Urbanucci

Last updated by author(s): Oct 9, 2024

Reporting Summary

Nature Portfolio wishes to improve the reproducibility of the work that we publish. This form provides structure for consistency and transparency in reporting. For further information on Nature Portfolio policies, see our Editorial Policies and the Editorial Policy Checklist.

Statistics

For all statistical analyses, confirm that the following items are present in the figure legend, table legend, main text, or Methods section.

n/a	Confirmed	
☐	☒	The exact sample size (n) for each experimental group/condition, given as a discrete number and unit of measurement
☒	☐	A statement on whether measurements were taken from distinct samples or whether the same sample was measured repeatedly
☐	☒	The statistical test(s) used AND whether they are one- or two-sided<br>*Only common tests should be described solely by name; describe more complex techniques in the Methods section.*
☒	☐	A description of all covariates tested
☐	☒	A description of any assumptions or corrections, such as tests of normality and adjustment for multiple comparisons
☐	☒	A full description of the statistical parameters including central tendency (e.g. means) or other basic estimates (e.g. regression coefficient) AND variation (e.g. standard deviation) or associated estimates of uncertainty (e.g. confidence intervals)
☐	☒	For null hypothesis testing, the test statistic (e.g. F, t, r) with confidence intervals, effect sizes, degrees of freedom and P value noted<br>*Give P values as exact values whenever suitable.*
☐	☒	For Bayesian analysis, information on the choice of priors and Markov chain Monte Carlo settings
☒	☐	For hierarchical and complex designs, identification of the appropriate level for tests and full reporting of outcomes
☐	☒	Estimates of effect sizes (e.g. Cohen's d, Pearson's r), indicating how they were calculated

Our web collection on statistics for biologists contains articles on many of the points above.

Software and code

Policy information about availability of computer code

Data collection	No software was used in data collection.
Data analysis	All code related to the manuscript is available at https://github.com/akiviaho/ST-prostate. Reproducible conda environments (separately for single-cell and spatial analyses) are available as environment files on github. Relevant package versions are also listed here: “anndata” v0.8.0, “matplotlib” v3.6.2, "ndpi2tiff" v1.8, “numpy” v1.22.4, “pandas” v1.5.2, “pydeseq2” v0.4.4, “scanpy” v1.9.1, “scib” v1.1.1, “scikit-learn” v1.1.3”, “scipy” v1.9.3, “scran” v1.18.5,“scrublet" v0.16.1 ,“scvi-tools” v0.16.1, “seaborn” v0.12.1, “spaceranger” v1.1.0, “statsmodels” v0.13.5, “squidpy” v1.2.3

For manuscripts utilizing custom algorithms or software that are central to the research but not yet described in published literature, software must be made available to editors and reviewers. We strongly encourage code deposition in a community repository (e.g. GitHub). See the Nature Portfolio guidelines for submitting code & software for further information.

Data

Policy information about availability of data

All manuscripts must include a data availability statement. This statement should provide the following information, where applicable:

- Accession codes, unique identifiers, or web links for publicly available datasets
- A description of any restrictions on data availability
- For clinical datasets or third party data, please ensure that the statement adheres to our policy

The study includes data generated from multiple cohorts, each collected with different ethical permits and written informed consent forms:

Discovery set includes samples from 1) Tampere University Hospital prospective sample collection (ethical permit R03203) 2) Tampere University hospital clinical trial (ethical permit 04078M, trial registration number NCT00293696), and UZ Leuven clinical trial “ARNEO” (trial registration number NCT03080116).
Validation cohort samples have been collected at St. Olav’s Hospital Trondheim, Norway (ethical permit 2017/576. Of the metastatic prostate cancer samples used in this study, three were acquired as part of the Johns Hopkins Medicine Institutional Review Board-approved (NA_00003925) Project to ELIminate lethal CANcer (PELICAN) from patients who provided written informed consent. One sample was acquired under Tampere University Hospital Ethics Committee approval R19074 from a patient who had provided written informed consent.

The processed spatial transcriptomics data presented in this study, excluding the validation cohort, have been deposited in the Gene Expression Omnibus (GEO) archive under accession identifier GSE278936 [https://www.ncbi.nlm.nih.gov/geo/query/acc.cgi?acc=GSE278936]. The raw sequencing data are available from the authors, but restrictions apply to the availability of these data. Data can be shared with qualified researchers in accordance with the conditions of ethical approvals and informed consent to use these data in research of prostatic diseases. All handling of these data must be in compliance with GDPR and other relevant data protection regulations upon completion of material transfer agreement with respective data controllers information. Data access requests will be processed at the earliest convenience. Data access will be granted for one year.

Validation cohort spatial transcriptomics data is available in the European Genome-Phenome Archive (EGA) under accession identifier EGAD50000000603[https://ega-archive.org/studies/EGAS50000000413]. The data is available under restricted access to ensure compliance with ethical and legal standards, including GDPR and approval from the Regional Committee for Medical Research in Norway. Access will be granted to researchers who meet these requirements, and they must sign a Data Access Agreement (DAA). To obtain access, contact the Data Access Committee (DAC) at NTNU, who will facilitate the process and provide access through the FEGA Norway node or HUNT Cloud once the DAA is completed. The DAA will be processed at the earliest convenience.

The single cell RNA-sequencing datasets used in this study are available on GEO under accession numbers GSE137829 [https://www.ncbi.nlm.nih.gov/geo/query/acc.cgi?acc=GSE137829], GSE141445 [https://www.ncbi.nlm.nih.gov/geo/query/acc.cgi?acc=GSE141445], GSE176031 [https://www.ncbi.nlm.nih.gov/geo/query/acc.cgi?acc=GSE176031], GSE185344 [https://www.ncbi.nlm.nih.gov/geo/query/acc.cgi?acc=GSE185344], and GSE181294 [https://www.ncbi.nlm.nih.gov/geo/query/acc.cgi?acc=GSE181294], the Sequence Read Archive (SRA) under accession number PRJNA699369 [https://www.ncbi.nlm.nih.gov/bioproject/PRJNA699369/] and on https://singlecell.broadinstitute.org/single_cell/study/SCP1244/ transcriptional-mediators-of-treatment-resistance-in-lethal-prostate-cancer. The bulk RNA-sequencing data used in this study is available for download on https://tcga.xenahubs.net (TCGA) and on https://www.cbioportal.org/study/summary?id=prad_su2c_2019 (SU2C-PCF). The remaining data are available within the Article, Supplementary Information or Source Data file. Source data are provided with this paper.

Research involving human participants, their data, or biological material

Policy information about studies with human participants or human data. See also policy information about sex, gender (identity/presentation), and sexual orientation and race, ethnicity and racism.

Reporting on sex and gender	Not applicable
Reporting on race, ethnicity, or other socially relevant groupings	Not reported
Population characteristics	Reported in Supplementary Table S1 (primary tumors) and Supplementary Table S10 (metastatic tumors).
Recruitment	Tampere cohort details found under clinical trial ID NCT00293696.<br>ARNEO cohort (UZ Leuven) details found under trial ID NCT03080116.<br>NTNU cohort prostate tissue specimens were collected from patients with untreated primary PCa who had given informed written consent before undergoing radical prostatectomy at St. Olav’s Hospital in Trondheim between 2008 and 2016.
Ethics oversight	The use of clinical material in Tampere was approved by the Ethics Committee of the Tampere University Hospital and the National Authority for Medicolegal Affairs.<br>Data generated in Universitaire Ziekenhuizen KU Leuven was collected as a part of the ARNEO trial (ClinicalTrials.gov ID NCT03080116).<br>Data generated in NTNU was generated from samples whose collection received approval from the regional ethical committee of Central Norway (identifier 2017/576) and adhered to both national and EU ethical regulations.<br>Three metastatic prostate cancer tumors were acquired as part of the Johns Hopkins Medicine Institutional Review Board-approved (NA_00003925) Project to ELIminate lethal CANcer (PELICAN) from patients who provided informed consent. One sample was acquired under Tampere University Hospital Ethics Committee approval R19074 from a patient who had provided written informed consent.

Note that full information on the approval of the study protocol must also be provided in the manuscript.

Field-specific reporting

Please select the one below that is the best fit for your research. If you are not sure, read the appropriate sections before making your selection.

☒ Life sciences ☐ Behavioural & social sciences ☐ Ecological, evolutionary & environmental sciences

For a reference copy of the document with all sections, see nature.com/documents/nr-reporting-summary-flat.pdf

Life sciences study design

All studies must disclose on these points even when the disclosure is negative.

Sample size	Sample size was not determined a priori.
Data exclusions	Single cells and spatial transcriptomics spots were excluded based on criteria disclosed in the manuscript (unique molecule counts, gene counts, etc.).
Replication	Lab protocols were documented and disclosed as part of the manuscript. All code related to the analyses were saved, documented and made publicly available to ensure reproducibility.
Randomization	No randomization was performed as part of the study.
Blinding	No blinding was performed.

Reporting for specific materials, systems and methods

We require information from authors about some types of materials, experimental systems and methods used in many studies. Here, indicate whether each material, system or method listed is relevant to your study. If you are not sure if a list item applies to your research, read the appropriate section before selecting a response.

Materials & experimental systems

n/a	Involved in the study	
☐	☒	Antibodies
☒	☐	Eukaryotic cell lines
☒	☐	Palaeontology and archaeology
☒	☐	Animals and other organisms
☐	☒	Clinical data
☒	☐	Dual use research of concern
☒	☐	Plants

Methods

n/a	Involved in the study	
☒	☐	ChIP-seq
☒	☐	Flow cytometry
☒	☐	MRI-based neuroimaging

Antibodies

Antibodies used	Antigens were stained with anti-PIGR 1:500 (Sigma-Aldrich, HPA012012, LOT#:000015811), anti-LTF 1:500 (Sigma-Aldrich, HPA059976, LOT#:000006001), anti-Pan Keratin (AE1/AE3/PCK26) (Roche, 760-2135, LOT#:F27094), anti-CP 1:150 (Sigma-Aldrich, HPA001834, LOT#:A43354), anti-CD66b 1:50 (Novus Biologicals, NB100-77808, LOT#:A-9), anti-CXCR2 1:2000 (Abcam, ab245982, LOT#:1000637-2), anti-CD45 1:100 (Cell Signaling Technology, #13917, LOT#:6) and anti-CD11b 1:100 (Cell Signaling Technology, #49420, LOT#:7). Detection was done with fluorescently labeled secondary antibodies Goat anti-Rabbit IgG (H+L) Highly Cross-Adsorbed Secondary Antibody, Alexa Fluor Plus 647 (Invitrogen, A32733, LOT#:XG349344), Goat anti-Mouse IgG (H+L) Cross-Adsorbed Secondary Antibody, Alexa Fluor 750 (Invitrogen, A21037, LOT#:2765637).
Validation	All antibodies used in this study were obtained from commercial sources and their functionality validated by their respective manufacturers. Each primary antibody was additionally tested in-house with positive control tissues suitable for the antigens of interest. The effect of antibody elution was tested for each staining separately by repeating the staining after antibody elution and comparing the results. For double-staining, secondary antibodies were tested for possible cross-reactivity.

Clinical data

Policy information about clinical studies

All manuscripts should comply with the ICMJEguidelines for publication of clinical research and a completedCONSORT checklist must be included with all submissions.

Clinical trial registration	A subset of samples used in the study were collected as part of clinical trials. The study at hand is not a clinical trial.<br>Tampere cohort details found under clinical trial IDs NCT00293696.<br>ARNEO cohort (UZ Leuven) details found under trial ID NCT03080116.
Study protocol	Study protocols are disclosed under their respective study identifiers, NCT00293696 (Tampere cohort), NCT03080116 (ARNEO / Leuven cohort).
Data collection	Data was not collected as a part of the current study, see NCT00293696, NCT03080116 for design of original the trials.
Outcomes	Outcome measures of the trial have not been considered in the current study, see NCT00293696, NCT03080116 for design of original the trials.

Plants

Seed stocks	*Report on the source of all seed stocks or other plant material used. If applicable, state the seed stock centre and catalogue number. If plant specimens were collected from the field, describe the collection location, date and sampling procedures.*
Novel plant genotypes	*Describe the methods by which all novel plant genotypes were produced. This includes those generated by transgenic approaches, gene editing, chemical/radiation-based mutagenesis and hybridization. For transgenic lines, describe the transformation method, the number of independent lines analyzed and the generation upon which experiments were performed. For gene-edited lines, describe the editor used, the endogenous sequence targeted for editing, the targeting guide RNA sequence (if applicable) and how the editor was applied.*
Authentication	*Describe any authentication procedures for each seed stock used or novel genotype generated. Describe any experiments used to assess the effect of a mutation and, where applicable, how potential secondary effects (e.g. second site T-DNA insertions, mosiacism, off-target gene editing) were examined.*
